# Non-destructive testing of mechanical components achieved by hybrid copper–iodide cluster†

**DOI:** 10.1039/d5ra00959f

**Published:** 2025-04-14

**Authors:** Rui-jia Dong, Ze-qi Wu

**Affiliations:** a Tangshan Polytechnic University Tangshan Hebei 063299 China zeqiwu@tsgzy.edu.cn; b Research and Development Center for Intelligent Manufacturing and Operation Application Technology of EMU Tangshan Hebei 063299 China

## Abstract

We synthesized a Cu_4_I_4_(3-picoline)_4_ cluster scintillator with high X-ray attenuation and a 89.25% photoluminescence quantum yield. *In situ* fabrication yielded screens showing a high light yield (60 617 photons per MeV), low detection limit (0.91 μGy_air_ s^−1^), and exceptional resolution (13 lp per mm). Non-destructive testing ability was demonstrated by imaging from plastic to metal.

## Abstract 2

A Cu_4_I_4_(3-pic)_4_ scintillator with high X-ray attenuation and PLQY was synthesized. The *in situ* fabricated screen achieved high light yield, low detection limit, and high spatial resolution, enabling efficient non-destructive testing.

## Graphical abstract

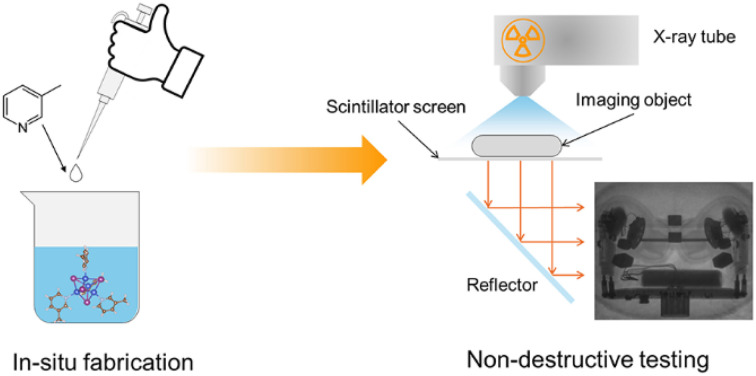

## Introduction

Since Wilhelm Conrad Röntgen discovered X-rays in 1895, radiography has undergone significant advancements, evolving into a critical technology for applications in medical diagnostics, security inspections, and industrial non-destructive testing due to its exceptional penetration capabilities.^1–3^ Currently, X-ray detection relies on two main approaches: direct detection and indirect detection. Indirect detection, which involves converting X-rays into visible light using scintillators, offers key advantages such as fast response times and easy integration with TFT or CMOS arrays, establishing it as the predominant technology on the market.^4,5^ As a result, scintillators play an indispensable role in X-ray detection. Traditional scintillation materials such as NaI:Tl and CsI:Tl have been extensively studied and applied in industrial settings for efficient X-ray scintillation.^3,6^ However, these materials often face significant limitations, including high-temperature fabrication, toxicity, and poor biocompatibility, underscoring the urgent need for alternative scintillation materials tailored to specific applications.^6,7^

Recently, metal halide complex-based scintillators have demonstrated outstanding scintillation performance.^5,8–11^ Their ease of synthesis, high luminescence efficiency, and cost-effective properties endow them with great potential. Among them, copper(i)–iodide (Cu–I) clusters have recently emerged as a promising class of scintillators, thanks to their high X-ray absorption, high light yield, and environmental friendliness.^9,12–15^ For instance, Tang *et al.* developed a flexible scintillator screen based on a Cu–I cluster, successfully achieving dynamic X-ray imaging of real objects.^16^ Similarly, Huang *et al.* synthesized monodisperse Cu–I cluster microcubes, which demonstrated exceptional stability under both moisture and X-ray exposure.^17^ Additionally, ultra-low detection limit was also achieved by Guo *et al.* based on a Cu–I cluster.^18^ These outstanding properties demonstrate that Cu–I clusters hold great promise for X-ray imaging applications in specialized or object-limited scenarios, such as medical diagnostics. As another important X-ray imaging filed, non-destructive testing is also facing growing and more advanced need. Unlike the relatively uniform nature of biological specimens analyzed in medical CT scans, industrial objects exhibit much greater diversity, with significant variations in material composition and quantity, especially in the field of smart manufacturing.^19^ Besides, industrial products are more and more integrated. These features demand scintillators to possess advantages including high light yield, high spatial resolution, and high energy resolution simultaneously.^1,20^ Cu–I cluster scintillator as a promising material, but their potential for non-destructive testing remains largely underexplored and in need of further development.

In this study, we synthesized the Cu–I cluster Cu_4_I_4_(3-picoline)_4_ [Cu_4_I_4_(3-pic)_4_] using a straightforward solution-based method. The single-crystal structure reveals dense atomic stacking, indicative of strong X-ray absorption potential. Under UV excitation, the cluster exhibits ^3^CC emission with a photoluminescence quantum yield (PLQY) of 89.25%. Additionally, an *in situ* synthesis approach was developed to produce highly emissive Cu_4_I_4_(3-pic)_4_ scintillator ink. The resulting scintillator screen demonstrated outstanding performance, including high light yield, excellent spatial resolution, and a low detection limit. Moreover, its successful application in the non-destructive testing of mechanical components underscores its potential to advance this critical field.

## Results and discussion

**Fig. 1:**
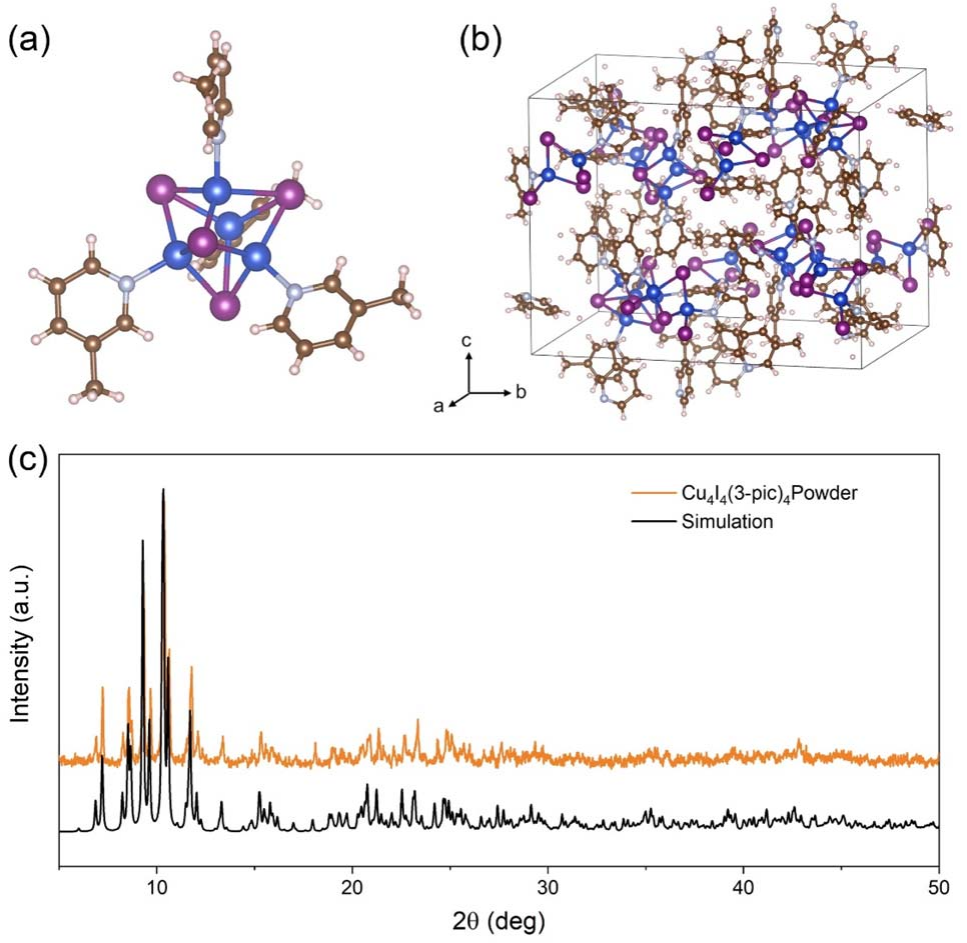
(a) Detailed view of Cu_4_I_4_(3-pic)_4_. (b) Unit cell packing diagram of Cu_4_I_4_(3-pic)_4_. (c) Experimental and simulated powder X-ray diffraction patterns of Cu_4_I_4_(3-pic)_4_.

We selected Cu_4_I_4_(3-pic)_4_ as the scintillator material due to its promising structural and functional properties. The molecular structure of the cluster, depicted in Fig. 1a, reveals that it crystallizes in the monoclinic space group *P*2_1_/*c*.^21^ Each cluster adopts a cubane-like geometry, where four copper and four iodine atoms alternately occupy the vertices of a distorted cubic framework. Within the Cu_4_I_4_ polyhedron, the longest Cu(i)–Cu(i) distances is 2.775 Å at 293 K, which is shorter than the sum of the van der Waals radii of two copper atoms (2.8 Å).^22^ This observation highlights the presence of strong Cu–Cu interactions, which play a critical role in cluster-centered (CC) emission and stabilizing the structure.^23,24^ Each copper atom is further coordinated by nitrogen atoms from 3-picoline ligands, contributing to both the structural integrity and the molecular stability of the cluster. The combination of the compact Cu_4_I_4_ core and the small-sized organic ligands facilitates dense molecular packing, as shown in Fig. 1b, which in turn leads to a high material density (2.213 g cm^−3^ at 293 K). This intrinsic density significantly enhances its X-ray attenuation and absorption capabilities, rendering it highly suitable for X-ray-related applications. The powder samples were synthesized using a solvent-based method, ensuring precise control over the formation of the desired crystalline phase. The purity of the resulting material was validated through powder X-ray diffraction analysis (Fig. 1c), where the experimental diffraction pattern showed excellent agreement with the simulated pattern, confirming the successful synthesis of the intended structure. Thermogravimetric analysis (Fig. S1†) of Cu_4_I_4_(3-pic)_4_ powder reveals a decomposition temperature of approximately 85 °C, confirming its stability under room temperature conditions. Furthermore, the Cu_4_I_4_(3-pic)_4_ powder sample demonstrated excellent air stability, showing no additional XRD peaks after 20 days of air exposure compared to the fresh sample (Fig. S2†).

**Fig. 2:**
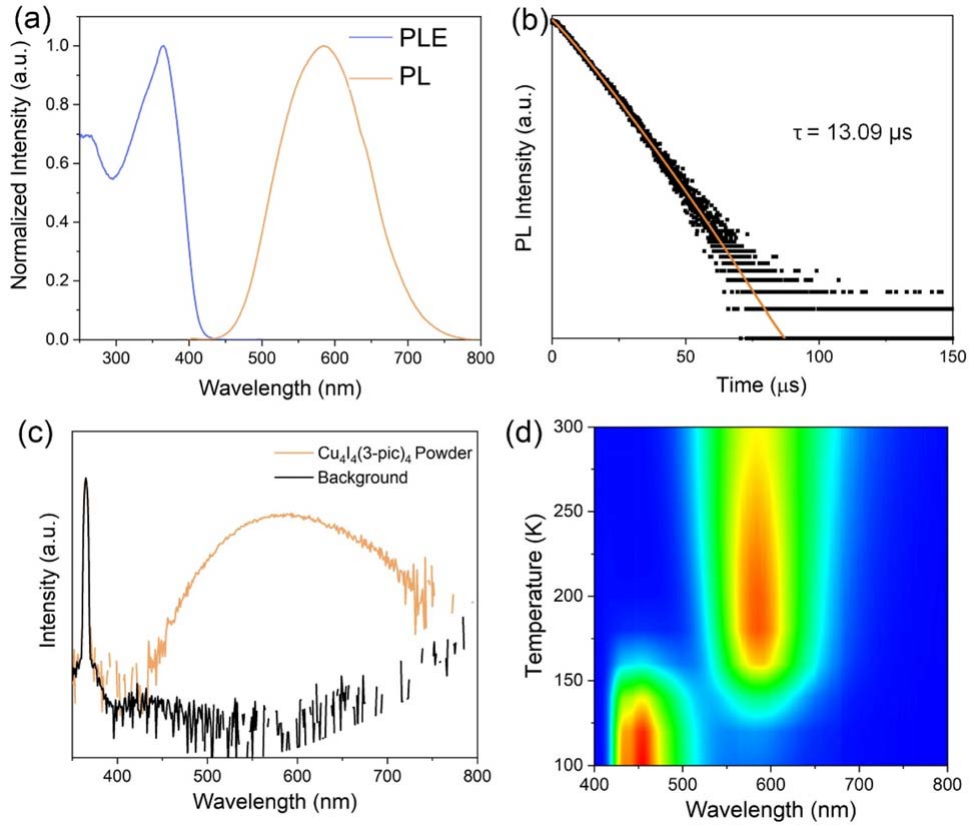
(a) Photoluminescence and photoluminescence excitation spectra. (b) Time-resolved PL decay under 365 nm excitation. (c) Photoluminescence quantum yield spectra. (d) Temperature dependent PL mapping.

**Fig. 3:**
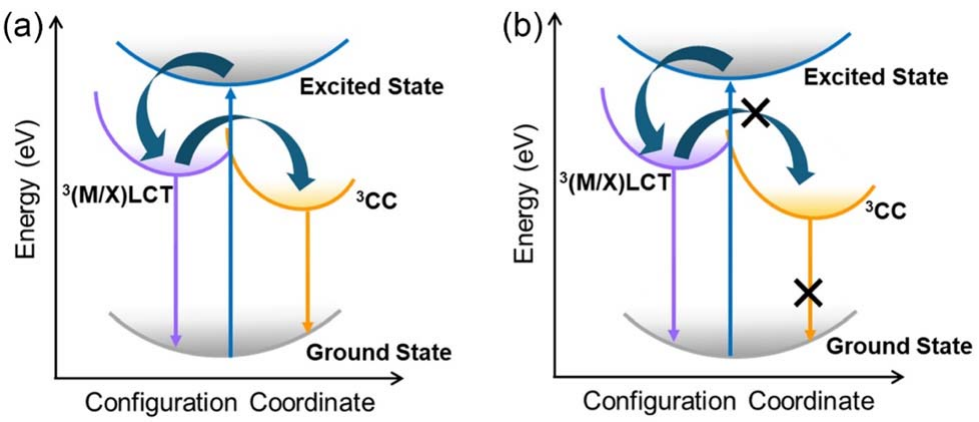
PL mechanism at (a) room temperature and (b) low temperature.

The photophysical properties of the material were systematically investigated using pure powder samples at room temperature. The photoluminescence (PL) and photoluminescence excitation (PLE) spectra are presented in Fig. 2a. A prominent and well-defined excitation peak is observed at 365 nm, consistent with values reported for analogous copper halide cluster compounds.^25–27^ Upon excitation at this wavelength, the material exhibits intense yellow photoluminescence, characterized by an emission maximum at 585 nm and a substantial Stokes shift of 220 nm. The PL emission profile features a full width at half maximum (FWHM) of 145 nm, indicative of a broad spectral emission. Time-resolved PL measurements were conducted by monitoring the emission at 585 nm, and the decay dynamics were fitted with a mono-exponential function (Fig. 2b). The resulting photoluminescence lifetime of 13.09 μs is notably long, providing strong evidence for triplet state emission characteristics. A high photoluminescence quantum yield (PLQY) up to 89.25% is also observed under excitation with 365 nm incident light (Fig. 2c), highlighting its great potential for photoluminescence applications. The temperature-dependent luminescence property and mechanism were studied by temperature-dependent PL measurement (excited at 365 nm light). Fig. 2d shows the temperature-dependent PL spectra of the Cu_4_I_4_(3-pic)_4_ powder from 100 K to 300 K. The overall changes of the emission peaks with the deceasing of temperature can be divided into 2 stages. In the first stage (300 K to 180 K), a single emission peak at 585 nm is observed, which intensifies as the temperature decreasing. At this stage, decreased temperature depressed the thermal vibration induced non-radiative recombination, leading to the enhancement of PL intensity. In the second stage (below 180 K), the 585 nm emission diminishes, and a new emission peak at approximately 450 nm emerges and grows stronger with further cooling. This transition results in a dramatic color shift, as shown in the CIE coordination (Fig. S3†), from yellow to deep blue. This luminescent thermochromism is attributed to the energy transfer between the triplet cluster-centered (^3^CC) states and triplet metal-and-iodine-to-ligand charge transfer [^3^(M + X)LCT] states which has been extensively documented in other 0D Cu_4_I_4_ clusters.^24,28–31^ At room temperature (Fig. 3a), excitons generated under UV excitation initially relax into the ^3^(M + X)LCT state but can also cross the energy barrier into the lower energy ^3^CC state. Thermal energy at higher temperatures facilitates this crossing, leading to a thermal equilibrium between the two states and resulting in emissions from ^3^CC states. As the temperature decreases, the reduced thermal energy inhibits state crossing, causing excitons to become trapped in the ^3^(M + X)LCT state as shown in Fig. 3b. This leads to a weakening of the ^3^CC-related low-energy emission at 585 nm and a strengthening of the ^3^(M + X)LCT-related high-energy emission at 450 nm.

**Fig. 4:**
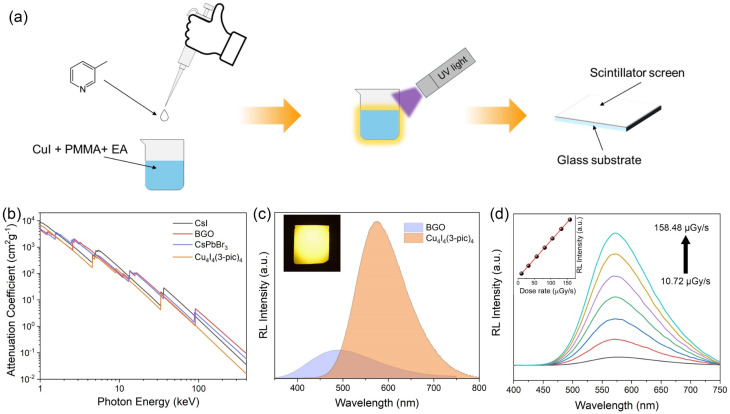
(a) Schematic illustration of the *in situ* synthesis of Cu_4_I_4_(3-pic)_4_ ink and preparation of scintillator screen. (b) Calculated X-ray absorption. (c) Steady-state radioluminescence (RL) spectra of Cu_4_I_4_(3-pic)_4_ scintillator screen and BGO. (Inset) Photograph of the Cu_4_I_4_(3-pic)_4_ scintillator screen under X-ray excitation. (d) RL intensity of Cu_4_I_4_(3-pic)_4_ screen as a function of the dose rate. (Inset) Linear fitting of dose rate-dependent RL intensity.

The Cu_4_I_4_(3-pic)_4_ ink was synthesized using an *in situ* fabrication method, as illustrated in Fig. 4a. Copper iodide and polymethyl methacrylate (PMMA) were mixed in ethyl acetate under vigorous stirring to ensure homogeneity, as shown in Fig. S4.† Subsequently, stoichiometric 3-picoline was added dropwise, immediately forming an emissive suspension that exhibited bright yellow light emission under UV excitation (Fig. S5 and S6†). The suspension was then carefully drop-cast onto a glass substrate and allowed to evaporate at room temperature for approximately 12 hours, resulting in a uniform PMMA film. The fabricated screen exhibits identical PL spectra with pure powder sample under 365 nm light excitation, exhibiting a yellow emission, as shown in Fig. S7.† To evaluate its X-ray attenuation properties, we compared the mass attenuation coefficient of Cu_4_I_4_(3-pic)_4_ to those of traditional scintillators and recently reported emerging materials. As shown in Fig. 4b, Cu_4_I_4_(3-pic)_4_ demonstrates a comparable of attenuation performance within the medical radiography energy range (1–400 keV).^8^ Under X-ray excitation, the Cu_4_I_4_(3-pic)_4_ scintillator screen exhibits the same emission as it does under 365 nm light (Fig. 4c inset). The light yield of the Cu_4_I_4_(3-pic)_4_ scintillator screen was determined based on steady-state radioluminescence (RL) spectra and linear attenuation efficiencies (Fig. S8†),^32–34^ giving a normalized light yield of 60 617 photons per MeV. The detailed calculation method is provided in the ESI.† Notably, this value surpasses that of some commercial scintillators, including BGO. (8900 ± 450 photons per MeV),^35^ LYSO:Ce (29 000 photons per MeV),^36^ and CsI (16 800 photons per MeV),^3^ and those of the previously reported Cu(i) based hybrids (Table S2†). The RL stability of the fabricated screen was confirmed after 30 minutes of continuous irradiation (Fig. S9†), showing only a 1.5% decrease in performance. Furthermore, a high degree linearity is demonstrated across the range of 10.72 μGy_air_ s^−1^ to 158.48 μGy_air_ s^−1^, enabling a reliable and predictable relationship between radiation intensity and scintillation output. A detection limit as low as 0.91 μGy_air_ s^−1^ was determined from the fitting curve at a signal-to-noise ratio of 3, as shown in Fig. 4d and its inset. This performance surpasses that of several other Cu–I cluster-based scintillators, as illustrated in Table S2.†

**Fig. 5:**
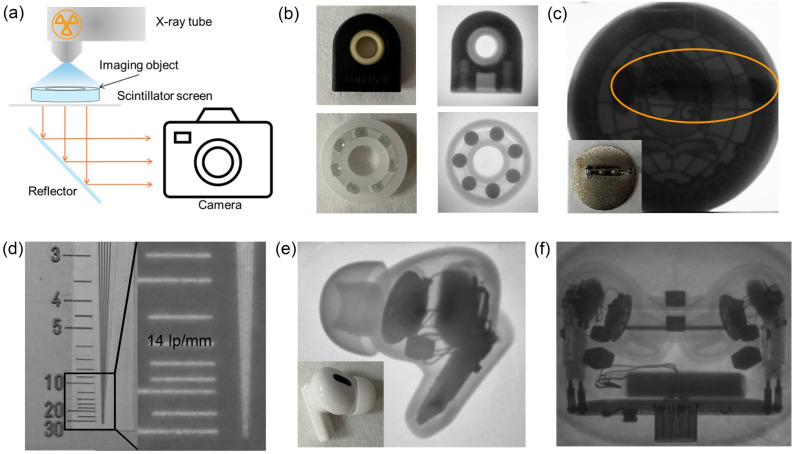
(a) Schematic diagram of X-ray imaging system. (b) Bearings under visible light (left) and X-ray (right). (c) Metal badge under X-ray. (Inset) Back of the metal badge under daylight. (d) Images of the standard line-pair card under daylight (left) and X-ray (right). (e) Images of the wireless earphone under X-ray and daylight (inset). (f) Images of the earphones in the charging case under X-ray.

Given the excellent scintillation performance of Cu_4_I_4_(3-pic)_4_, we further explored its application in X-ray radiography based on large-sized scintillator screen (Fig. S10†). A custom-built X-ray imaging system, shown in Fig. 5a, was used to evaluate the non-destructive testing capabilities of the Cu_4_I_4_(3-pic)_4_ scintillator screen. Various mechanical components, ranging from plastic to metal, including two bearings of different materials and a metal badge, were tested. As demonstrated in Fig. 5b, the internal channel of a plastic sliding bearing is clearly visible, highlighting the ability of Cu_4_I_4_(3-pic)_4_ to effectively image low X-ray attenuation materials. The clear contrast between the polypropylene plastic and the glass balls within the rolling bearing further underscores the high energy resolution of the scintillator screen. This level of resolution is crucial for mechanical non-destructive testing, where components may vary in material composition. Additionally, an aluminum alloy badge was used to assess the imaging performance of the Cu_4_I_4_(3-pic)_4_ scintillator screen for metallic objects (Fig. S11†). As shown in Fig. 5c, the pin behind the badge was also successfully imaged. These results demonstrate the suitability of the Cu_4_I_4_(3-pic)_4_ scintillator screen for non-destructive inspection in complex mechanical environments. Spatial resolution is another key property for X-ray non-destructive testing as crack can be tiny. We compared the spatial resolution of the fabricated scintillator screen with a standard lead line pair card. As shown in Fig. 5d, it achieved a remarkable spatial resolution of 14 lp per mm, giving a high distinguishing ability up to 71 μm. The modulation transfer function (MTF) of the scintillator screen is also presented in Fig. S12,† from which a spatial resolution of ∼13 lp per mm can be extracted at a MTF = 0.2. This value is basically consistent with the value we obtained from the X-ray images of a standard X-ray test line pair card. A wireless earphone was utilized to showcase the practical non-destructive inspection capabilities of the system. As illustrated in Fig. 5e, the high-quality X-ray images reveal clear details of the electronic components, with even the electric wires distinctly visible. Furthermore, the charging case was examined with earphones enclosed within the case, their internal structures are clearly distinguishable. Additionally, the charging circuit beneath the metal shell at the bottom of the case is also imaged. The ability of the Cu_4_I_4_(3-pic)_4_ scintillator screen to produce high-quality imaging of both plastic and metal components in a single shot highlights its excellent energy resolution, further demonstrating the effectiveness of the system for non-destructive testing in industrial applications.

## Conclusions

In summary, this study demonstrates the exceptional potential of Cu_4_I_4_(3-pic)_4_ as a high-performance scintillator for X-ray non-destructive testing. The unique cubane-like molecular structure, featuring strong Cu–Cu interactions and dense molecular packing, ensuring it high photoluminescence efficiency and high X-ray attenuation. High quality scintillator screen was fabricated based on an *in situ* fabrication method for Cu_4_I_4_(3-pic)_4_ ink. The screen demonstrated remarkable light yield (60 617 photons per MeV^−1^), low detection limit (0.91 μGy_air_ s^−1^), and high spatial resolution (13 lp per mm). Furthermore, the scintillator screen also shows capability of resolving intricate details in various materials from plastic to metal. All these benefits establish Cu_4_I_4_(3-pic)_4_ as a promising material for advanced X-ray imaging and industrial non-destructive testing.

## Data availability

The data supporting this article have been included as part of the ESI.†

## Conflicts of interest

There are no conflicts to declare.

## Supplementary Material

RA-015-D5RA00959F-s001
