# Supplementary material for: Non-destructive testing of mechanical components achieved by hybrid copper–iodide cluster
Source: RSC Adv. 2025 Apr 14;15(15):11639–44. doi: 10.1039/d5ra00959f (PMC11995392; doi:10.1039/d5ra00959f)
Supplement: RA-015-D5RA00959F-s001 [file RA-015-D5RA00959F-s001.pdf]

Supplementary Information (SI) for RSC Advances.
This journal is © The Royal Society of Chemistry 2025

Supporting Information

Experimental Section

Chemical Preparations: Copper iodide (CuI, 99.9%), ethanol (99%), 3-picoline (3-pic, 99%), ethyl acetate, and poly(methyl methacrylate) (PMMA, average Mw 2,000,000) were purchased commercially and used without further purification.

Synthesis of Cu_4I_4(3-pic)$_4$ Powder: CuI (1 mmol, 190 mg) was dispersed in 10 mL of ethanol and stirred vigorously to form a uniform suspension. Subsequently, 0.9 mmol (84 mg) of 3-picoline was added dropwise to the suspension. White powder formed immediately. The Cu_4I_4(3-pic)$_4$ powder was separated by centrifugation (7000 rpm for 5 minutes) and dried under vacuum for 12 hours.

Preparation of Scintillator Screen: PMMA (100 mg) was dissolved in 2 mL of ethyl acetate under stirring and heated at 60°C for 6 hours. After cooling to room temperature, CuI (1 mmol, 190 mg) was added under vigorous stirring, followed by the dropwise addition of 1 mmol of 3-picoline. The resulting scintillator ink, with white powder suspended uniformly in the solvent, was obtained after 3 minutes of stirring. The scintillator screen was fabricated by drop-casting the ink evenly onto a glass substrate and allowing it to evaporate at room temperature for approximately 12 hours.

Characterizations: X-ray diffraction (XRD) measurements were performed using a Cu Kα X-ray tube (40 kV, 40 mA) with a D8 Advance (Bruker) X-ray diffractometer. Thermogravimetric analysis (TGA) was conducted with a heating rate of 10 °C/min from 30°C to 600°C under a nitrogen flow (PerkinElmer Instruments, Diamond TG/DSC6300). Photoluminescence excitation (PLE), photoluminescence (PL), and radioluminescence (RL) measurements were carried out using a Horiba spectrometer. Photoluminescence quantum yield (PLQY) was measured on the same system with an additional integrating sphere. Time-resolved photoluminescence decay curves were obtained using a fluorescence spectrometer (FLS-1000) with a 372 nm nano-LED as the excitation source. For RL and X-ray imaging tests, an X-ray tube (W target, TUB00153-9 series, MOXTEK) was used as the excitation source, operating at 50 kV with a current of 30 μA for RL spectra and 50 μA for imaging. The X-ray dose rate used for detection limit test was measured using a commercial X-ray dosimeter from Radcal company. High-resolution images were captured using a commercial digital camera (Nikon D7100) with an exposure time of 30 seconds for the metal badge and 15 seconds for the other items.

Calculation: The light yield of the Cu_4I_4(3-pic)$_4$ scintillator screen was determined using the following equation:

$$LY_{sc} = \frac{LY_{BGO} \times \frac{PC_{SC}/AE_{SC}}{PC_{BGO}/AE_{BGO}}}{}$$

Where the LY_{sc} is the light yield of our scintillator screen, LY_{BGO} is the reported light yield of BGO,[1] PC_{SC} and PC_{BGO} are the photon counts of scintillator screen and BGO obtained by integrating the steady-state RL spectra. AE_{SC} and $AE_{BGO}$are the linear attenuation efficiencies at a sample thickness of 500 μm for the entire X-ray photon energy range (from 0 to 50 keV), which were calculated based on a reported method.[2] The X-ray output spectrum of our tube was calculated following a previously established method[3], and the linear attenuation efficiencies of BGO and Cu_4I_4(3-pic)$_4$ were obtained from the XCOM database of the National Institute of Standards and Technology.

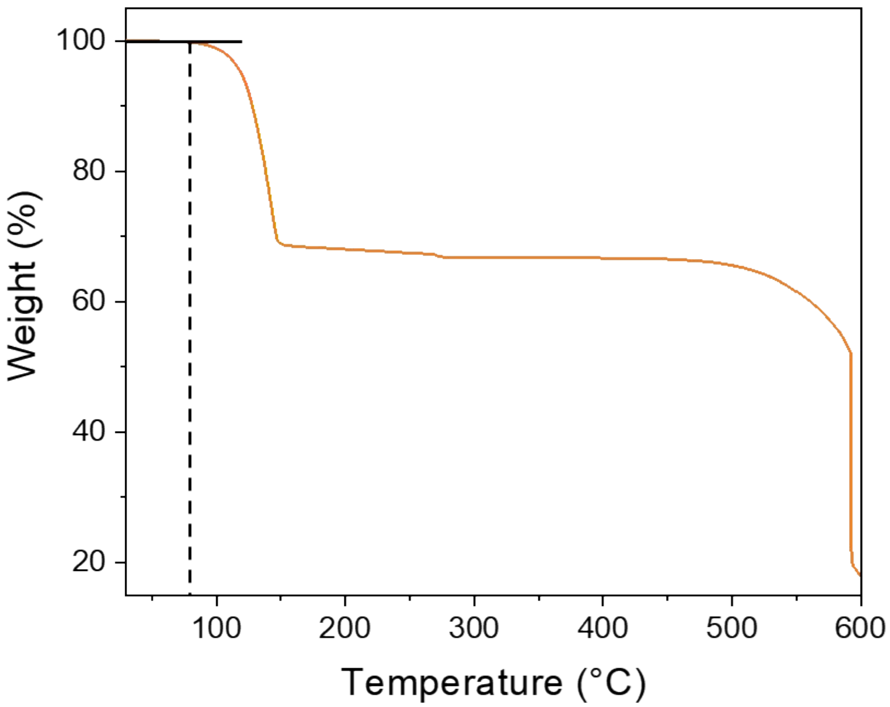

Figure S1. Thermogravimetric analysis of $Cu_4I_4(3\text{-pic})_4$ powder sample from 30 to 600 °C.

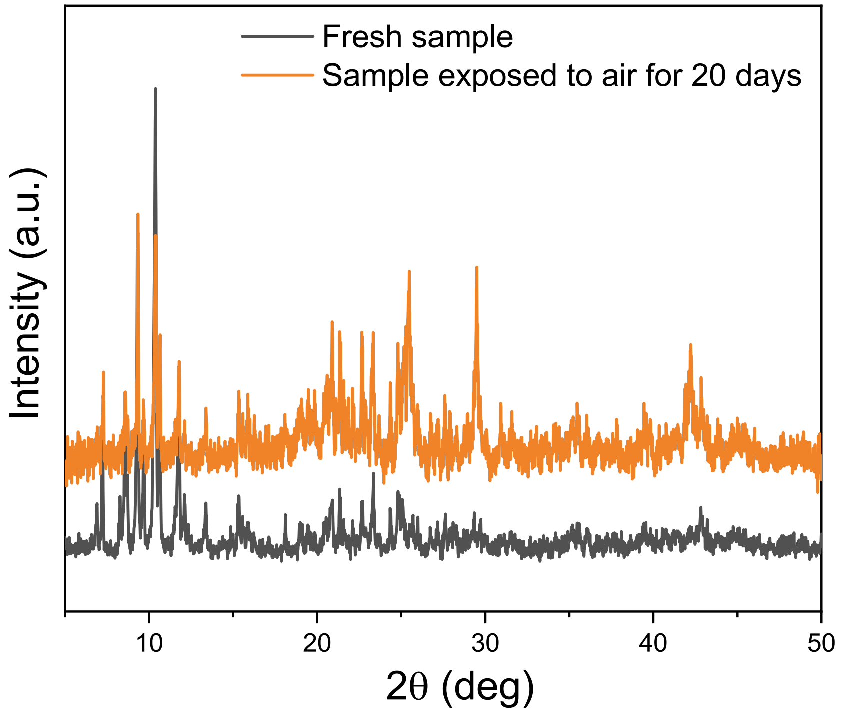

Figure S2. PXRD patterns of fresh sample and exposed to air for 20 days.

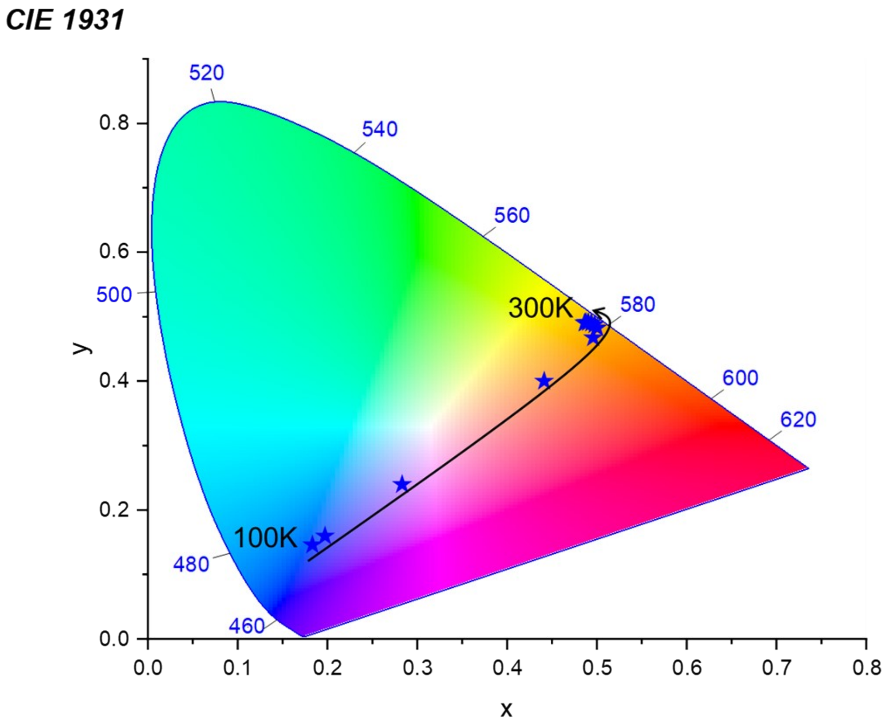

Figure S3. Temperature-dependent CIE 1931 coordination of Cu_4I_4(3-pic)$_4$ from 100 to 300 K.

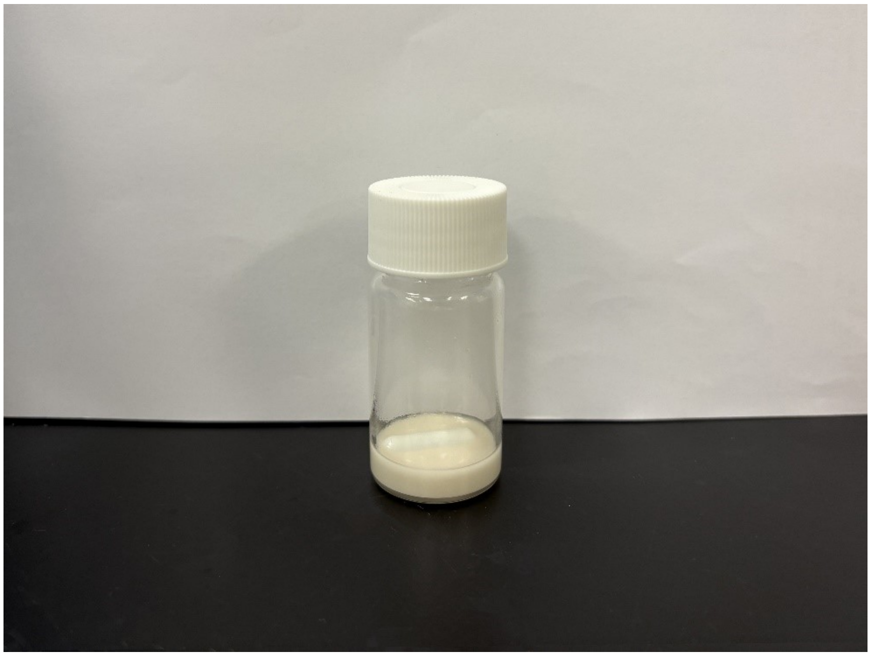

Figure S4. Photo of CuI/PMMA in ethyl acetate.

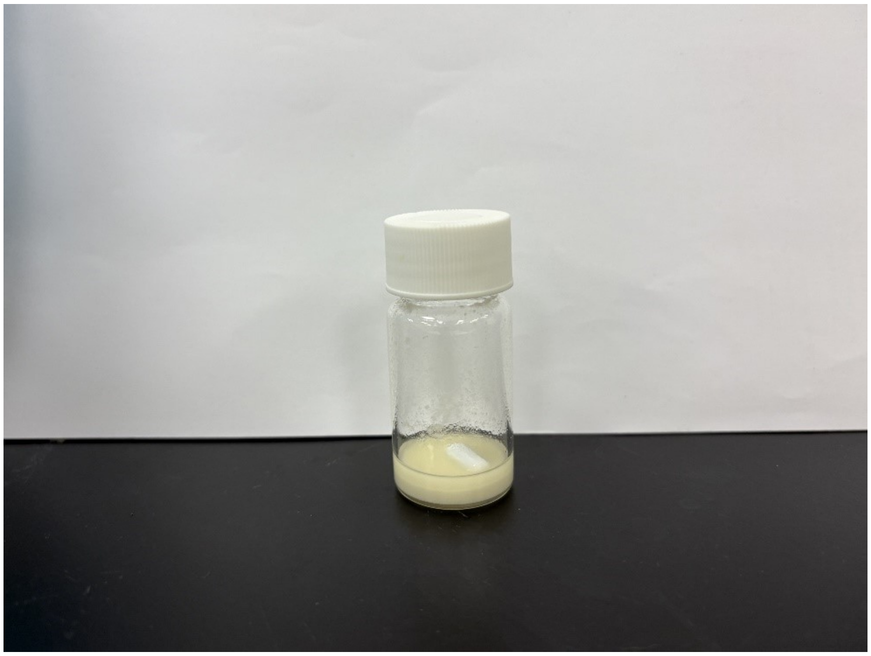

Figure S5. Photo of $Cu_4I_4(3\text{-pic})_4$ scintillator ink under sunlight.

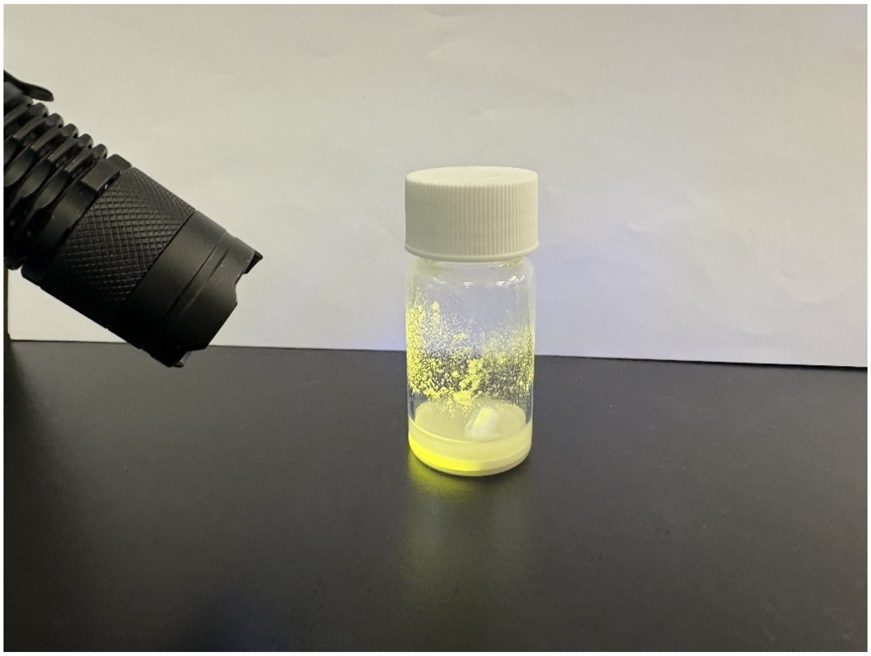

Figure S6. Photo of $Cu_4I_4(3\text{-pic})_4$ scintillator ink under UV-light.

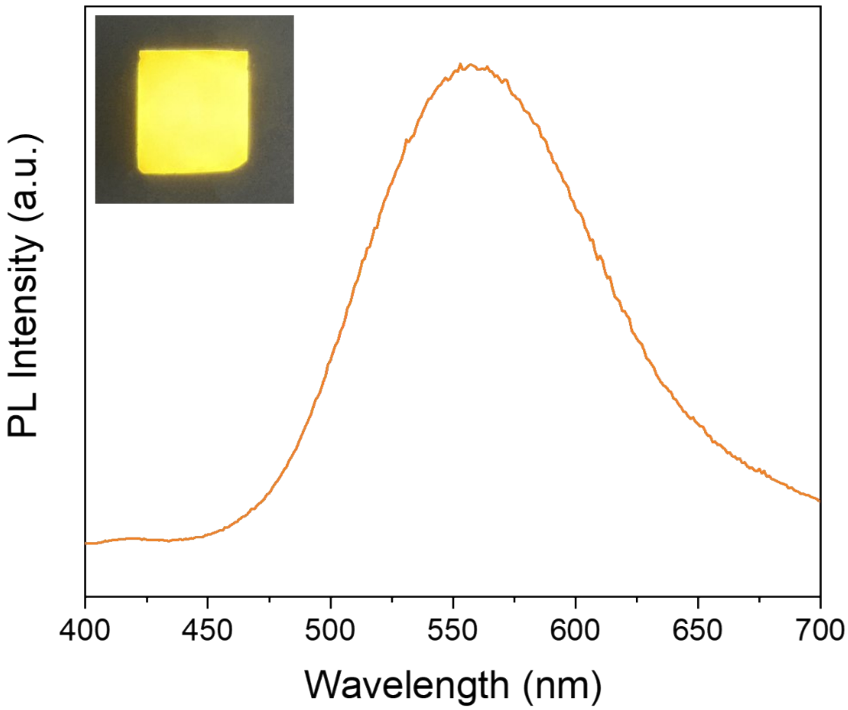

Figure S7. Photoluminescence spectra of Cu_4I_4(3-pic)$_4$ scintillator screen at ambient condition. Inset: photograph of the Cu_4I_4(3-pic)$_4$ scintillator screen under 365 nm light excitation.

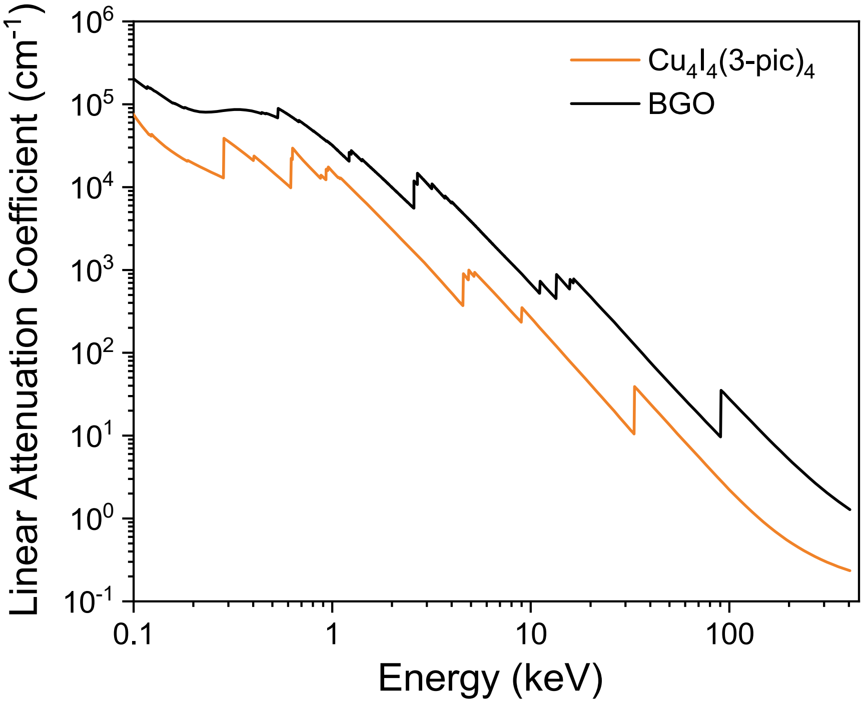

Figure S8. Calculated X-ray linear attenuation coefficient of Cu_4I_4(3-pic)$_4$ and BGO.

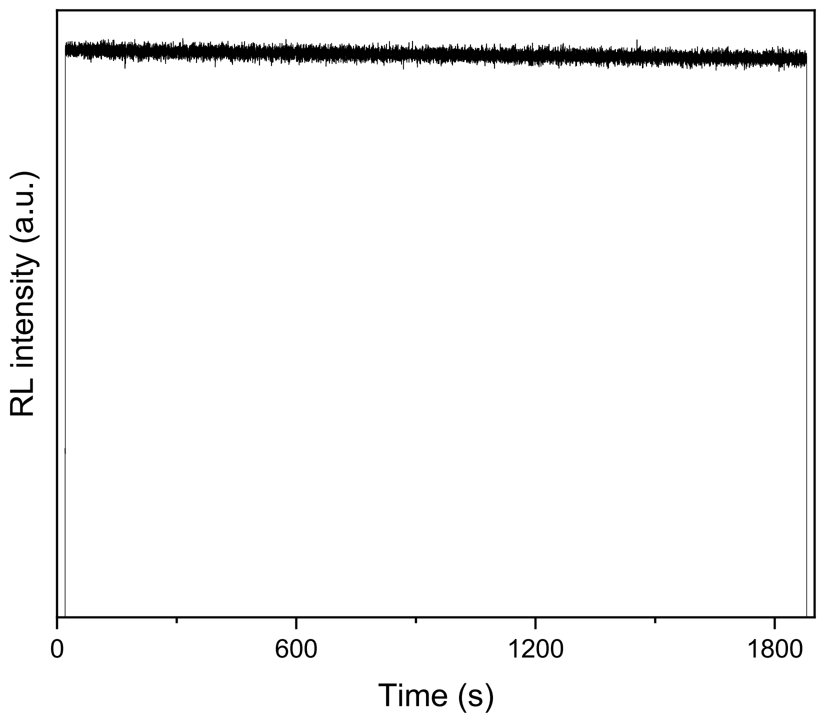

Figure S9. RL emission intensity under constant X-ray irradiation.

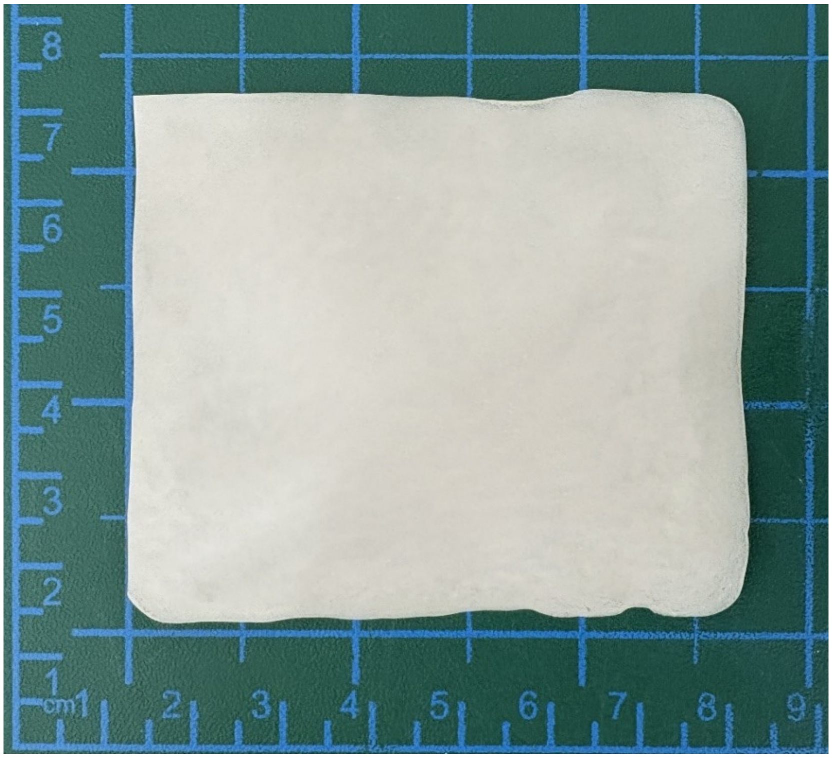

Figure S10. Photo of large size $Cu_4I_4(3\text{-pic})_4$ scintillator screen.

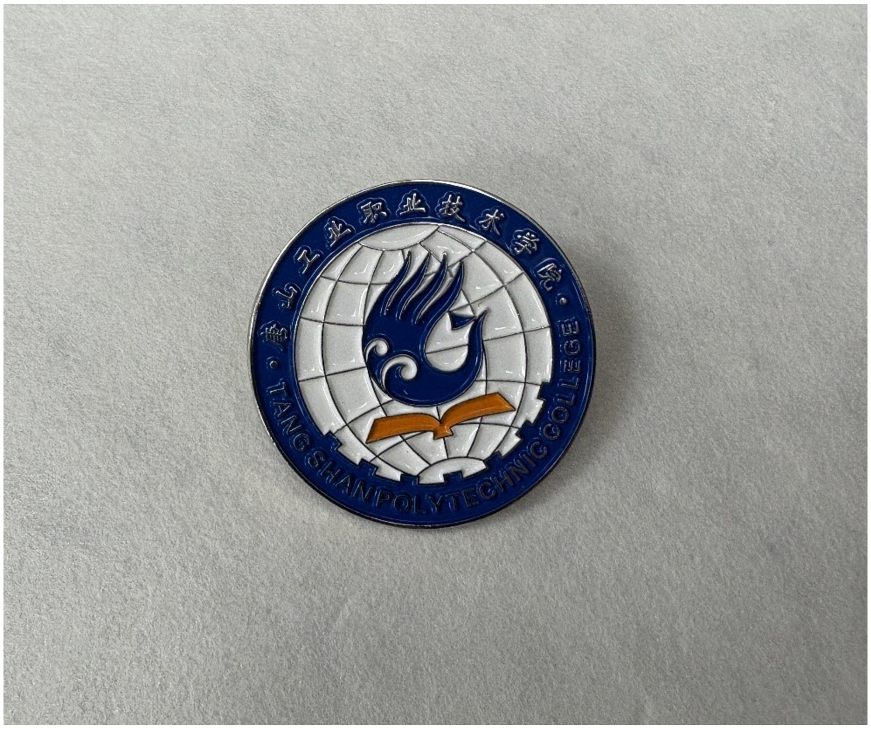

Figure S11. The front of aluminum alloy badge used for X-ray imaging.

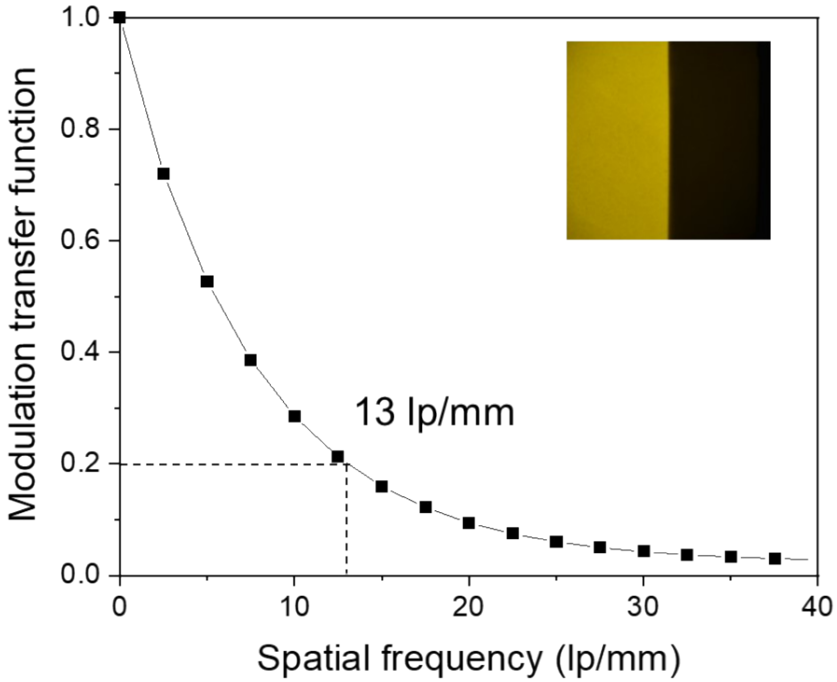

Figure S12. Spatial resolution of the X-ray imaging system, characterized by the modulation transfer function under X-ray exposure (50 kV 30 μA) and corresponding location of the target.

Table S1 Crystal data and structure refinement for Cu_4I_4(3-pic)$_4$.

Empirical formula	$C_{24}H_{28}Cu_4I_4N_4$
Temperature/K	293
Crystal system	monoclinic
Space group	$P2_1/c$
a/Å	15.1190(4)
b/Å	24.5144(6)
c/Å	18.3911(5)
α/°	90
β/°	92.495(1)
γ/°	90
Volume/Å^3	6809.89
Z	8
ρ_{calc}g/cm^3	2.213
F(000)	4224
Radiation	Mo Kα (λ = 0.71073)
Θ range for data collection/°	1.35 to 25
Refinement method	Full-matrix least-squares on F^2
Goodness- of - fit on F^2	0.938
Index ranges	100 ≤ h ≤ -100, 100 ≤ k ≤ -100, 100 ≤ l ≤ -100
Final R indexes [I>=2σ (I)]	R_1 = 0.0401, wR_2 = 0.0401
R indices (all data)	R_1 = 0.0843, wR_2 = 0.0932

Table S2. Light yield, detection limit and spatial resolution of Cu_4I_4(3-pic)$_4$ scintillator screen compared to other reported Cu-I-based scintillators.

Compounds	Light yield (photons MeV^{-1})	Detection limit (nGy$_{air}$ s^{-1})	Spatial resolution (lp/mm)
**Cu_4I_4(3-pic)$_4$**	**60617**	**910**	**14**
$(Cu_3I_6)_4(C_{16}H_{34}N_2)_6$[4]	17508	1140	
(4-bzpy)$_4Cu_4I_4$[5]	60948	239700	5
β-$(MePh_3P)_2CuI_3$[6]	16193	47.97	
CuI(PPh3)$_2$(3-Mepy)[7]	28385 ± 1335	43.8	9.8

$PDACuI_3$[8]	8100	288	16
Tb-Cu_4I_4[9]	29379	45.2	12.6
CuI(py)[10]	2.52 times stronger than that of BGO	55	20

References

1. I. Holl, E. Lorenz and G. Mageras, *IEEE Trans. Nucl. Sci*, 1988, **35**, 105−109.
2. W. Ma, Y. Su, Q. Zhang, C. Deng, L. Pasquali, W. Zhu, Y. Tian, P. Ran, Z. Chen, G. Yang, G. Liang, T. Liu, H. Zhu, P. Huang, H. Zhong, K. Wang, S. Peng, J. Xia, H. Liu, X. Liu and Y. M. Yang, *Nat Mater*, 2022, **21**, 210-216.
3. W. Pan, H. Wu, J. Luo, Z. Deng, C. Ge, C. Chen, X. Jiang, W.-J. Yin, G. Niu, L. Zhu, L. Yin, Y. Zhou, Q. Xie, X. Ke, M. Sui and J. Tang, *Nat. Photonics*, 2017, **11**, 726-732.
4. Q. Wang, H. Li, J. Fan, Z. Zhou, H. Tong, J. Zhu, W. Liu and G. Ouyang, *Inorganic Chemistry Frontiers*, 2024, **11**, 7399-7406.
5. Q. Kong, X. Jiang, Y. Sun, J. Zhu and X. Tao, *Inorganic Chemistry Frontiers*, 2024, **11**, 3028-3035.
6. J. L. Qi, Y. Guo, J. Wu, Q. F. Huang, J. J. Xu, S. F. Yan, W. Liu and S. P. Guo, *Angew. Chem. Int. Ed. Engl.*, 2024, DOI: 10.1002/anie.202407074, e202407074.
7. X. Liu, Y. Jiang, F. Li, X. Xu, R. Li, W. Zhu, J. Ni, C. Ding, S. Liu and Q. Zhao, *Adv. Optical Mater.*, 2022, **11**.
8. Y. Du, L. Ma, Z. Yan, J. Xiao, K. Wang, T. Lin, X. Han and D. Xia, *Inorg. Chem.*, 2023, **62**, 11350-11359.
9. X. Liu, R. Li, X. Xu, Y. Jiang, W. Zhu, Y. Yao, F. Li, X. Tao, S. Liu, W. Huang and Q. Zhao, *Adv. Mater.*, 2023, **35**, e2206741.
10. W. Zhao, Y. Wang, Y. Guo, Y. D. Suh and X. Liu, *Adv. Sci. (Weinh)*, 2022, DOI: 10.1002/advs.202205526, e2205526.
